# Comorbidity in an Older Population with Type-2 Diabetes Mellitus: Identification of the Characteristics and Healthcare Utilization of High-Cost Patients

**DOI:** 10.3389/fphar.2020.586187

**Published:** 2020-11-30

**Authors:** Inmaculada Guerrero-Fernández de Alba, Valentina Orlando, Valeria M. Monetti, Sara Mucherino, Antonio Gimeno-Miguel, Olga Vaccaro, Maria João Forjaz, Beatriz Poblador Plou, Alexandra Prados-Torres, Gabriele Riccardi, Enrica Menditto

**Affiliations:** ^1^ EpiChron Research Group, Aragon Health Sciences Institute (IACS), IIS Aragón, Miguel Servet University Hospital, Zaragoza, Spain; ^2^ Health Services Research on Chronic Patients Network (REDISSEC), ISCIII, Madrid, Spain; ^3^ CIRFF, Center of Pharmacoeconomics and Drug utilization Research, Department of Pharmacy, University of Naples Federico II, Naples, Italy; ^4^ Department of Pharmacy, University of Naples Federico II, Naples, Italy; ^5^ Department of Clinical Medicine and Surgery, University of Naples Federico II, Naples, Italy; ^6^ National Centre of Epidemiology, Institute of Health Carlos III and REDISSEC, Madrid, Spain

**Keywords:** drug utilization, diabetes cost, multimorbidity, real-world data, type-2 diabetes mellitus

## Abstract

**Objectives: **Little is known about the specific comorbidities contributing to higher costs in patients with type-2 diabetes mellitus (T2DM), particularly in older cases. We aimed to evaluate the prevalence, type, and cost of comorbidities occurring in older T2DM patients versus older non-T2DM patients, and the factors associated with high cost (HC) T2DM patients.

**Methods:** Retrospective cohort study using information from the Campania Region healthcare database. People aged ≥65 years who received ≥2 prescriptions for antidiabetic drugs were identified as “T2DM patients.” Comorbidities among T2DM and non-T2DM groups were assessed through the RxRiskV Index (modified version). T2DM individuals were classified according to the total cost distribution as HC or “non-high cost.” Two sub-cohorts of HC T2DM patients were assessed: above 90th and 80th percentile of the total cost. Age- and sex-adjusted logistic regression models were created.

**Results:** Among the T2DM cohort, concordant and discordant comorbidities occurred significantly more frequently than in the non-T2DM cohort. Total mean annual cost per T2DM patient due to comorbidities was €7,627 versus €4,401 per non-T2DM patient. Among T2DM patients identified as being above 90th and 80th percentiles of cost distribution, the total annual costs were >€19,577 and >€2,563, respectively. The hospitalization cost was higher for T2DM cases. Strongest predictors of being a HC T2DM patient were having ≥5 comorbidities and renal impairment.

**Conclusion:** HC patients accrued >80% of the total comorbidities cost in older T2DM patients. Integrated care models, with holistic and patient-tailored foci, could achieve more effective T2DM care.

## Introduction

In the past three decades, the prevalence of type-2 diabetes mellitus (T2DM) has increased dramatically worldwide to become an important healthcare concern (World Health Organization, 2016; Zimmet, 2017). In 2018, T2DM prevalence in Italy was estimated as 6.2% of the total population and ∼approximately 67% of T2DM patients are aged ≥65 years (ARNO diabetes observatory, 2019). The resulting health and economic impacts go beyond the condition itself because T2DM patients are subject to disabling complications, such as cardiovascular or renal diseases (Van Dieren et al., 2010; Schinner, 2011), and incur very high costs to the Italian National Health Service (Hutter et al., 2010; O’Shea et al., 2013; Lehnert et al., 2011). Piette and Kerr (2006) identified two types of T2DM-associated comorbidities. They developed a framework in which chronic conditions are conceptualized as concordant (pathophysiologic profile or management plan similar to T2DM) or discordant (pathophysiologic condition and disease-management plan not directly related to T2DM). With the increasing burden of T2DM comorbidity, the aforementioned framework has served as a basis to improve T2DM management and to study the impact of multimorbidity (Calderón-Larrañaga et al., 2014; Lin et al., 2018; Aga et al., 2019). Indeed, a recent study in Spain demonstrated that the coexistence of mental and other discordant comorbidities in T2DM patients may increase the use of healthcare resources significantly (Calderón-Larrañaga et al., 2015). The economic impact of T2DM-related comorbidities cannot be overlooked due to its multiple incurred expenditures, either due to a higher prevalence of hospitalization and visits to the general practitioner, or due to an increase in the number of drugs used (American Diabetes Association, 2018). Given that disproportion, other studies have identified high-cost (HC) patients in different diseases, such as T2DM or acute coronary syndrome (Rais et al., 2013; Wodchis et al., 2016).

Characterizing the effects of different comorbidities in HC patients may represent an opportunity to implement interventions addressing patient-centered care models to care better for T2DM patients with complex disease.

Scholars have examined the economic impact of coexisting chronic disease in T2DM patients based on counting the number of diseases, but less is known about which specific types of comorbidities contribute primarily to higher costs (Kerr et al., 2007). Moreover, several scholars have investigated the entire T2DM population rather than focusing on the older T2DM population, which usually presents a higher percentage of patients with complex diseases (Illario et al., 2015; Illario et al., 2016). Hence, we aimed to evaluate: 1) the prevalence, type and cost of comorbid chronic diseases occurring in T2DM patients older than 65 years compared with those of a non-T2DM population older than 65 years; 2) which factors are associated at a higher cost in T2DM patients.

## Materials and Methods

### Study Design

A retrospective cohort study was carried out using information collected routinely in a healthcare database in the Campania Region of Southern Italy. The Campania Region Database (CaReDB) includes patient-level demographic information, electronic records of outpatient pharmacy dispensing, and hospital discharge for ∼6 million residents of a well-defined region in Italy (∼10% of the Italian population). Data are tracked longitudinally via de-identified and unique patient numbers. For the purpose of this analysis, data from 1 January 2017 through 31 December 2018 were used. CaReDB is complete and includes validated data used in previous drug utilization studies (Iolascon et al., 2013; Casula et al., 2014; Orlando et al., 2016; Guerriero et al., 2017; Russo et al., 2018; Orlando et al., 2020). The characteristics of CaReDB are described in Supplementary Table S1.

### Study Population

The study population consisted of people aged ≥65 years who had received medication dispensation according to CaReDB between 1 January and 31 December 2017 (enrollment period). Individuals with T2DM were identified by selecting those who had received ≥2 prescriptions for antidiabetic drugs alone or in combination with any type of insulin, as a proxy for disease diagnosis (Moreno et al., 2019). Individuals who did not receive any antidiabetic agent during the study period were used as the comparator group for the analysis and are referred to as “non-T2DM” cohort.

### Patient Characteristics

Comorbidity in T2DM group and non-T2DM group was assessed using a modified version of the RxRiskV Index, a validated pharmaceutical-based comorbidity index derived from dispensation data using Anatomical Therapeutic Chemical classification codes (O’Shea et al., 2013; Pratt et al., 2018). The RxRiskV Index was adapted for our study by including updated Anatomical Therapeutic Chemical codes for medications licensed in Italy currently (Supplementary Table S2). Individuals were classified as having one of the conditions listed in the RxRiskV Index if they received at ≥2 consecutive dispensations of a drug for treatment of a specific class of disease. Comorbidities were classified as concordant or discordant on expert opinions’ consensus taking as reference the definition of chronic comorbidities of T2DM by Piette and colleagues (Piette and Kerr, 2006). T2DM was excluded from the list because it was the disease of interest in the present study. Individuals were categorized by sex and stratified into three age groups; 65–69, 70–74, and ≥75 years. The number of medications dispensed, and all-cause hospital admissions were estimated in T2DM group and non-T2DM group.

### Outcome

The total cost, related to all comorbidities, was calculated as the sum of medical costs and dispensed drugs costs for both T2DM and non-T2DM group in 2018 (follow-up period). The total healthcare cost included drug expenses to treat the comorbidities selected in the RxRisk Index, excluding those attributed directly to T2DM. The hospitalization cost included the cost of all-cause hospitalization incurred in each group. In accordance with recent studies (Meyers et al., 2014; Wammes et al., 2018; Nelson et al., 2019), primary care visits were proxied using prescriptions. Therefore, each prescription is counted as a visit. T2DM patients were classified according to the distribution of the total cost as HC or “non-high cost” (NHC) individuals. We created two sub-cohorts of HC T2DM individuals: patients whose costs were above the 90th percentile of the total cost; patients whose costs were above the 80th percentile of the total cost. We also created two sub-cohorts of NHC T2DM individuals, with costs below the 90th and 80th percentile of the total healthcare cost. Costs were expressed in euros at time of analyses.

### Statistical Analysis

The median level of comorbidity, interquartile range, and prevalence of the most common comorbid conditions defined by the RxRiskV Index were assessed in those with T2DM and those not suffering from T2DM. Descriptive analyses of patient characteristics were calculated as frequencies and proportions, and the use of healthcare services as the mean number and median number of prescriptions, primary care visits, and hospitalizations. Differences between people suffering from and not suffering from T2DM were compared using chi-square test for categorical variables, and the Wilcoxon–Mann–Whitney test or Student’s t-test for numerical variables. The annual average cost of drugs and hospitalizations by sex, age, type, and number of comorbidities was estimated in the T2DM group and non-T2DM group.

Age- and sex-adjusted logistic regression models were employed to examine the association between the comorbidity prevalence rates and T2DM status (T2DM vs non-T2DM group). A regression model for each comorbidity with a prevalence ≥5% in T2DM group was created. The adjusted odds ratios (ORs) were calculated and displayed with their respective 95% confidence intervals (95% CI).

Among T2DM patients, the sub-cohorts formed by HC patients and NHC patients were characterized in terms of demographic variables (sex and age), comorbidity score (categorized as greater or less than five comorbidities), type of comorbidity (concordant, discordant, or both), prevalence of each comorbidity, as well as the use and cost of healthcare services. To assess predictors of being a HC patient with T2DM, two logistic regression models, for the >90th and >80th percentile of the total cost, were performed, respectively. The demographic variables included as independent variables were sex (reference: female) and age (reference: 65–69 years). The clinical variables included were comorbidity score (reference <5 comorbidities), presence of concordant/discordant comorbidities, and receipt of insulin (reference: use of insulin). Data management was carried out with a Microsoft SQL server v2018 (Penton Media, Loveland, CO, United States). Analyses were undertaken with SPSS v17.1 (IBM, Armonk, NY, United States) and *p* < 0.05 was considered significant.

## Results

A total of 1,011,671 people aged >65 years were included in our study. Among them, 197,992 (19.6%) received ≥2 prescriptions for antidiabetic drugs and were identified as the T2DM cohort.

The age (mean ± standard deviation, SD) was 74.8 ± 6.7 years for the T2DM cohort and 74.7 ± 7.5 years for the non-T2DM cohort. Most individuals (88.5%) had at least one of the comorbidities of interest, increasing up to 97.6% in the T2DM group. The median number of comorbid conditions was 5 (Interquartile range, IQR: 3–7) among T2DM patients and 3 (IQR: 1–6) among non-T2DM individuals. Significant differences between T2DM and non-T2DM cohorts were recorded in the mean number of prescriptions (38.2 vs. 24.8), primary care visits (16.5 ± 9.8 vs. 11.2 ± 9.2), percentage of patients who had more than one hospitalization (20.4% vs. 13.8%) and percentage of patients with >3 days of stay in hospital as an inpatient (12.3% vs. 8.0%). Characteristics of T2DM and non-T2DM cohorts are shown in Table 1.

**TABLE 1:** Characteristics of T2DM and non-T2DM individuals over 65 years of age.

	T2DM cohort *N* = 197,992	NON-T2DM cohort *N* = 813,679	*p*-value
Sex	—	—	—
Female (%) Male (%)	53.1 46.9	57.4 42.6	<0.001 <0.001
Mean age (SD)	74.8 (6.7)	74.7 (7.5)	<0.001
65–69 years (%)	26.8	31.5	<0.001
70–74 years (%)	26.2	24.0	<0.001
≥75 years (%)	47.0	44.5	<0.001
Comorbid conditions, median number (IQR)	5 (3–7)	3 (1–6)	<0.001
Number of prescriptions	—	—	—
Mean (SD)	38.2 (22.8)	24.8 (20.3)	<0.001
Median (IQR)	36 (21–52)	21 (9–37)	—
Primary care visits	—	—	—
Mean (SD)	16.5 (9.8)	11.2 (9.2)	<0.001
Median (IQR)	15 (10–22)	10 (4–16)	—
Hospital admission	—	—	—
Had ≥1 hospitalization (%)	20.4	13.8	<0.001
Mean (SD)^a^	1.6 (1.1)	1.4 (0.9)	<0.001
Median (IQR)^a^	1 (1–2)	1 (1–2)	—
Inpatient days (total)	—	—	—
>3 days, %	12.3	8.0	<0.001
Median (IQR)^a^	9 (5–17)	8 (5–15)	—

T2DM, Type 2 diabetes mellitus; SD, Standard deviation; IQR, Interquartile range.

a Among patients with ≥1 hospitalization.

Table 2 shows prevalence of comorbidities and ORs adjusted by sex and age in T2DM and non-T2DM groups. The vast majority of comorbidities occurred significantly more frequently among T2DM patients. Individuals in T2DM cohort were significantly more likely to have hyperlipidemia as a comorbid condition than people in the non-T2DM cohort (OR 3.42, 95% CI, 3.38–3.45), followed by hyperuricemia/gout (2.49, 2.45–2.53), cerebrovascular disease (2.82, 2.80–2.85) and ischemic heart disease/angina (2.17, 2.11–2.22). Among discordant comorbidities, gastro-oesophageal reflux disease (GORD) and peptic ulcer was the most prevalent comorbidity in T2DM group and was more prevalent than that in the non-T2DM group (OR 2.22, 95% CI, 2.20–2.24). Mental health conditions were most frequently detected in T2DM cohort: epilepsy (OR 1.92, 1.88–1.96); depression (OR 1.24, 1.22–1.26). However, two conditions recorded a significantly lower prevalence in the T2DM cohort when compared with non-T2DM cohort: osteoporosis (3.8% and 5.0%, respectively) and corticosteroid-responsive diseases (6.2% and 9.2%, respectively), defined as inflammatory conditions generally treated with mineralocorticoids and glucocorticoids.

**TABLE 2:** Chronic comorbidities with ≥5% prevalence in the study population with and without type-2 diabetes mellitus (T2DM).

	T2DM cohort (%)	NON-T2DM cohort (%)	Or (95% CI)^a^
Concordant conditions	—	—	—
Ischaemic heart disease/Angina	5.0	2.3	2.166 (2.112–2.221)
Cerebrovascular disease	50.1	26.1	2.824 (2.796–2.853)
Arrhythmia	7.1	6.0	1.157 (1.135–1.180)
Renal disease	35.6	26.6	1.493 (1.478–1.509)
Heart disease	52.8	35.9	1.991 (1.971–2.011)
Hyperlipidemia	60.4	30.7	3.417 (3.382–3.452)
Hyperuricemia/Gout	12.9	5.5	2.491 (2.450–2.532)
Hypertension	42.7	31.5	1.625 (1.609–1.642)
Discordant conditions	—	—	—
Coagulation disorders	11.7	8.6	1.382 (1.360–1.404)
Benign prostatic hypertrophy	15.4	12.7	1.141 (1.124–1.159)
Chronic airways disease	16.3	13.6	1.205 (1.189–1.221)
GORD and peptic ulcer	67.9	48.7	2.220 (2.197–2.243)
Glaucoma	8.4	4.9	1.776 (1.742–1.809)
Hypothyroidism	7.2	5.5	1.431 (1.403–1.459)
Osteoporosis	3.8	5.0	0.804 (0.783–0.824)
Inflammatory/Pain	25.9	21.1	1.340 (1.325–1.355)
Pain (treated with opiates)	5.3	3.4	1.605 (1.568–1.642)
Corticosteroid-responsive diseases^b^	6.2	9.2	0.657 (0.644–0.670)
Depression	10.4	8.7	1.238 (1.217–1.258)
Epilepsy	7.6	4.1	1.917 (1.879–1.956)

GORD, Gastro-oesophageal reflux disease; OR, Odds ratio; CI, Confidence interval.

a Adjusted by sex and age.

b Defined as inflammatory conditions generally treated with mineralocorticoids and glucocorticoids.

The total mean annual cost per patient in the T2DM cohort due to comorbidities was €7,627 (95% CI: 7,512–7,741) and €4,401 (4,359–4,443) in the non-T2DM cohort (Table 3A), and the difference in cost (“cost ratio”) was 1.73. The hospitalization cost contributed to ∼90% of total cost in both groups, with a significant difference between the T2DM group and non-T2DM group (cost ratio: 1.77). The greatest difference between the two groups was for the hospitalization cost related to micro/macrovascular complications (cost ratio: 2.38); this represented ∼40% of the total hospitalization cost in the T2DM group.

The total mean cost attributable to comorbidities between T2DM and non-T2DM people, stratified by sex and age, showed differences that decreased with age (Table 3B). Concordant and discordant comorbidities showed a higher total cost in T2DM group, with a cost ratio of 1.89 and 1.25 for concordant and discordant comorbidities, respectively. Furthermore, as the number of comorbidities increased, the average cost per patient also increased, with around 30% higher costs in the T2DM cohort (Table 3C).

**TABLE 3A:** Total mean annual cost (€) of chronic comorbidities among cohorts with and without type-2 diabetes (T2DM).

	T2DM cohort Mean (CI)	NON-T2DM cohort Mean (CI)	Cost ratio
Total	7,627.0 (7,512.3–7,741.5)	4,401.4 (4,359.5–4,443.3)	1.73
Drug cost^a^	615.9 (611.8–620.0)	438.8 (437.1–440.5)	1.40
Micro/Macro vascular hospitalization cost	2,849.1 (2,775.7–2,922.4)	1,197.2 (1,173.1–1,221.3)	2.38
Other causes hospitalization cost	4,161.9 (4,083.5–4,240.3)	2,765.3 (2,733.8–2,797.0)	1.51
Total hospitalization cost^b^	7,011.0 (6,897.0–7,125.0)	3,962.6 (3,920.9–4,004.2)	1.77

CI, Confidence Interval.

a Includes all drugs for the treatment of comorbidities.

b Includes all hospitalizations due to both complications and comorbidities.

**TABLE 3B:** Total mean annual cost (€) of chronic comorbidities among cohorts with and without T2DM stratified by sex and age.

	T2DM cohort Mean (CI)	NON-T2DM cohort Mean (CI)	Cost ratio
Sex	—	—	—
Female	6,716.4 (6,573.6–6,859.2)	3,903.1 (3,852.2–3,954.0)	1.72
Male	8,669.4 (8,485.9–8,852.8)	5,091.8 (5,021.0–5,162.5)	1.70
Age group	—	—	—
65–69 years	6,651.9 (6,438.9–6,864.8)	3,226.4 (3,161.0–3,291.8)	2.06
70–74 years	7,873.7 (7,638.9–8,108.5)	4,387.9 (4,300.3–4,475.5)	1.79
≥75 years	8,033.8 (7,867.8–8,199.7)	5,174.8 (5,108.8–5,240.9)	1.55

CI, Confidence Interval.

**TABLE 3C:** Total mean annual cost (€) of chronic comorbidities among cohorts with and without T2DM stratified by type and number of comorbidities.

	T2DM cohort Mean (CI)	NON-T2DM cohort Mean (CI)	Cost ratio
Concordant comorbidities	1,965.4 (1,853.1–2,077.6)	1,041.5 (1,009.3–1,073.7)	1.89
Discordant comorbidities	1,466.4 (1,338.7–1,594.1)	1,177.6 (1,140.0–1,215.2)	1.25
Number of comorbidities^a^	—	—	—
1	618.1 (563.1–673.0)	452.8 (439.3–466.2)	1.37
2	1,524.8 (1,439.2–1,610.5)	1,132.8 (1,104.8–1,160.8)	1.35
3	2,564.4 (2,445.7–2,683.1)	1,979.4 (1,934.5–2,024.4)	1.30
4	3,841.8 (3,690.7–3,993.0)	3,086.7 (3,018.6–3,154.7)	1.24
≥5	10,867.6 (10,687.8–11,047.3)	8,124.4 (8,031.7–8,217.2)	1.34

CI, Confidence Interval.

a Incremental cost for each comorbidity in addition to T2D.

**FIGURE 1:**
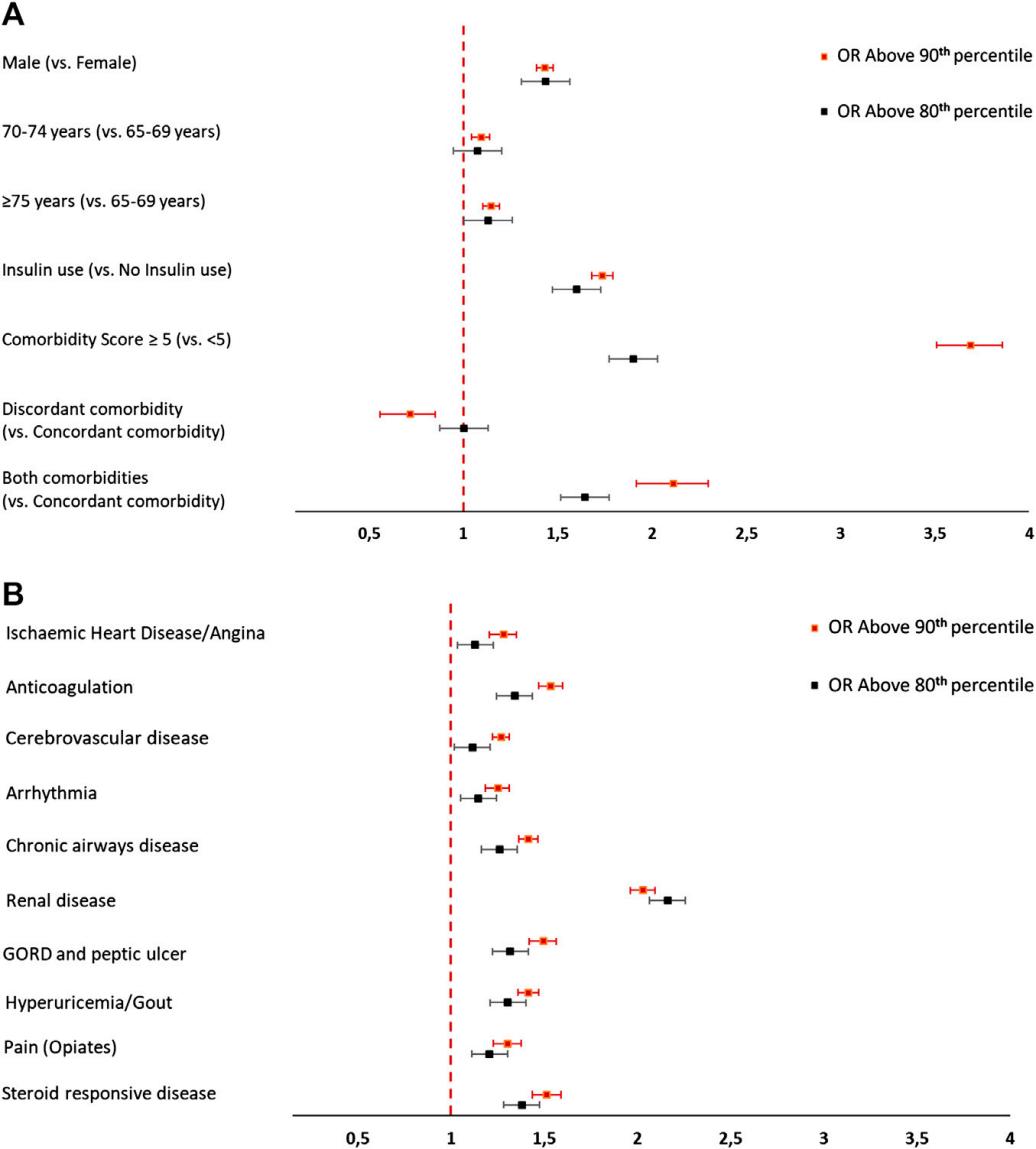
**(A)** Characteristics of high-cost and non-high-cost T2DM patients. **(B)** Proportions of comorbidities in high-cost and non-high-cost T2DM patients.

Among T2DM patients (*N* = 197,992), 19,319 were identified as being above the 90th percentile of cost distribution (more than €19,577), and 38,639 as being above the 80th percentile (more than €2,563) (Table 4). The annual total cost of the T2DM population included in this study amounted to approximately €1.47 billion. The HC patients (above the 90th percentile) accrued costs of more than €1.20 billion, which represented ∼80% of the total cost. HC patients (above the 80th percentile) accrued costs of more than €1,39 billion, which represented ∼90% of the total cost related to this population. Differences were found between HC patients and NHC individuals in terms of age and sex distribution (Table 4). The cost increased with the comorbidity score. More than 85% of HC patients above the 90th percentile and >75% of HC patients above the 80th percentile had ≥5 chronic comorbidities. Higher use of insulin was recorded for HC patients (39.3 and 35.1%, respectively, for people above the 90th percentile and above the 80th percentile of costs), than for NHC individuals (24.4 and 23.6%, respectively for subjects above the 90th percentile and below the 80th percentile of costs). The most common conditions in individuals above the 90th and 80th percentile of the total cost were: GORD and peptic ulcer (86.5 and 79.9%, respectively), hyperlipidemia (70.3 and 65.1%, respectively) and heart disease (70.0 and 61.5%, respectively) (Figure 1). With regard to variables in use of healthcare services, HC patients had a significantly higher number (mean ± SD) of annual visits in primary care than that for NHC individuals (22.3 ± 10.4 vs. 16.4 ± 9.3 in people above and below the 90th percentile; 20.4 ± 10.5 vs. 16.1 ± 9.2 in individuals above and below the 80th percentile) and higher number (mean ± SD) of prescriptions (54.8 ± 23.8 vs. 37.2 ± 21.6 in people above or below the 90th percentile; 49.4 ± 24.6 vs. 36.4 ± 21.1 in individuals above or below the 80th percentile). More than half of people above the 90th percentile was hospitalized at least twice per year (this figure was almost 100% if we measured those who were hospitalized at least once). Only 1.7% of people below the 90th percentile recorded a number of hospitalizations per year >2, and ∼12% were hospitalized at least once a year. Looking at number of inpatient days subjects above the 90th percentile of costs recorded 18.4 [19.9] mean number [SD] vs 8.4 [7.1] in patients below the 90th percentile. The largest difference between T2DM patients above the 90th percentile and 80th percentile of the total-cost distribution and T2DM patients below the 90th percentile and 80th percentile of total-cost distribution was the hospitalization cost, which was markedly higher among HC patients than NHC patients in both groups (Table 4).

**TABLE 4:** Characteristics of high-cost and low–cost T2DM patients.

	T2DM cohort			
	High–cost	Non-high–cost	High–cost	Non-high–cost
	Patients	Patients	Patients	Patients
	Above 90th percentile	Below 90th percentile	Above 80th percentile	Below 80th percentile
	*N* = 19,319	*N* = 173,870	*N* = 38,639	*N* = 154,550
Sex, *N* (%)	—	—	—	—
Female	9,244 (47.8)	93,879 (54.0)	18,473 (47.8)	84,650 (54.8)
Male	10,075 (52.2)	79,991 (46.0)	20,166 (52.2)	69,900 (45.2)
Age, *N* (%)	—	—	—	—
65–69 years	4,271 (22.1)	46,739 (26.9)	9,123 (23.6)	41,887 (27.1)
70–74 years	5,062 (26.2)	45,641 (26.3)	10,109 (26.2)	40,594 (26.3)
≥75 years	9,986 (51.7)	81,490 (46.9)	19,407 (50.2)	72,069 (46.6)
Age mean (SD)	75.3 (6.4)	74.7 (6.8)	75.1 (6.5)	74.8 (6.8)
Comorbidity score	—	—	—	—
Mean (SD)	7.3 (2.4)	5.2 (2.5)	6.4 (2.6)	5.1 (2.5)
Median (IQR)	7 (6–9)	5 (3–7)	6 (5–8)	5 (3–7)
Comorbidity score, *N* (%)	—	—	—	—
<5 comorbidities	2,447 (12.7)	72,237 (41.5)	9,285 (24.0)	65,399 (42.3)
≥5 comorbidities	16,872 (87.3)	101,633 (58.5)	29,354 (76.0)	89,151 (57.7)
Insulin use (%)	7,600 (39.3)	42,496 (24.4)	13,569 (35.1)	36,527 (23.6)
Primary-care visits	—	—	—	—
Mean (SD)	22.3 (10.4)	16.4 (9.3)	20.4 (10.5)	16.1 (9.2)
Median (IQR)	21.0 (15–28)	15 (10–21)	19 (13–26)	15 (10–21)
Number of prescriptions	—	—	—	—
Mean (SD)	54.8 (23.8)	37.2 (21.6)	49.4 (24.6)	36.4 (21.1)
Median (IQR)	53 (39–68)	35 (21–50)	47 (32–63)	34 (20–49)
Hospital admission, *N* (%)	—	—	—	—
≥1	19,299 (99.9)	21,090 (12.1)	37,139 (96.1)	3,250 (2.1)
≥2	10,195 (52.8)	2,898 (1.7)	12,966 (33.6)	127 (0.1)
Inpatient days	—	—	—	—
>3 days, *N* (%)	15,748 (81.5)	8,312 (4.8)	23,646 (61.2)	414 (0.3)
Mean (SD)	18.4 (19.9)	8.4 (7.1)	14.9 (17.2)	8.5 (11.2)
Median (IQR)	11 (6–23)	7 (4–10)	9 (5–17)	6 (4–9)
Drug cost (€)	—	—	—	—
Mean (95% CI)	1,119.0 (1,091.8–1,146.1)	560.0 (556.7–563.3)	1,036.6 (1,019.0–1,054.2)	510.7 (508.3–513.1)
Hospitalization cost (€)	—	—	—	—
Mean (95% CI)	60,843.3 (6,0044.4–61,642.3)	1,029.6 (1,013.8–1,045.4)	34,938.9 (34,461.7–35,416.1)	28.7 (27.6–29.8)
Total cost (€)	—	—	—	—
Mean (CI)	61,962.3 (61,161.9–62,762.7)	1,589.6 (1,573.4–1,605.8)	35,975.6 (35,497.5–36,453.6)	539.5 (536.9–542.0)

CI, Confidence interval; SD, standard deviation; IQR, interquartile rang.

**FIGURE 2:**
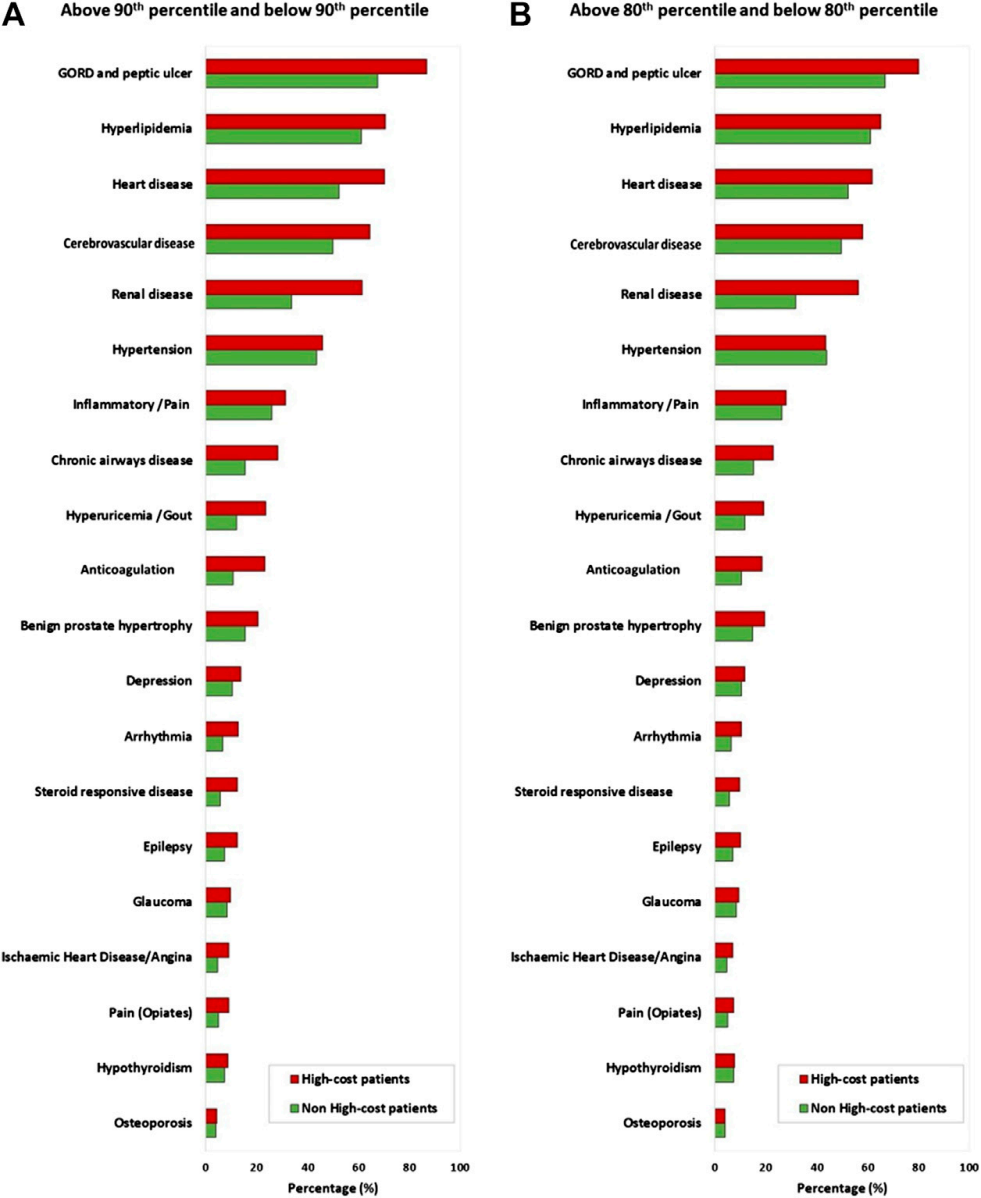
**(A)** Predictors of being a high-cost T2DM patient. **(B)** Predictive comorbidities of being a high-cost T2DM patient. Notes: Both multivariate regression models are adjusted for sex and age.

Logistic regression (Figures 2A) showed that a person in the oldest age group (≥75 years) was more likely to be a HC T2DM patient (in the top-10 or top-20 decile of the cost distribution) than a person in the younger age group. Men were ∼43% more likely to be HC patients (in both groups) compared with women. The strongest predictor of being a HC T2DM patient (in the top-10 decile or top-20 decile of the cost distribution) was having ≥5 comorbidities (top-10: OR 3.65, 95% CI, 3.49–3.82; top-20: 1.94, 1.88–1.99).

In addition, insulin use was associated with becoming a HC patient (top-10: OR 1.74, 95% CI, 1.68–1.79; top-20: 1.61, 1.57–1.65) and having discordant and concordant comorbidities was a strong predictor of being a HC patient in the top-10 decile (2.76, 2.42–3.15) or in top-20 decile of the cost distribution (1.76, 1.66–1.88) (Figures 2A). Furthermore, among concordant comorbidities, renal impairment was the strongest predictor of being a HC patient (top-10: 2.03, 1.96–2.09; top-20: 2.15, 2.10–2.20) followed by hyperuricemia/gout (90th: 1.41, 1.36–1.47; 80th: 1.30, 1.26–1.35). Among discordant comorbidities, GORD and peptic ulcer (90th: 1.49, 1.42–1.56; 80th: 1.31, 1.27–1.35) and corticosteroid-responsive diseases (90th: 1.51, 1.43–1.59; 80th: 1.38, 1.32–1.44) showed a higher likelihood of a person becoming a HC patient (Figures 2B).

## Discussion

We investigated prevalence and cost of comorbid conditions, concordant and discordant with T2DM, in a population of 1,011,671 individuals aged >65 years. We also examined and quantified differences in healthcare costs between HC and NHC T2DM patients identifying the type and number of comorbidities in the top-10 and top-20 percentiles of the cost distribution.

T2DM presented a higher prevalence of comorbidities and related cost compared with that in non-T2DM population. Our results are in accordance with data from Ireland and Australia (Caughey et al., 2010; O’Shea et al., 2013). Overall, cardiovascular diseases represented a substantial part of concordant comorbidities in the T2DM group and non-T2DM group, but the prevalence of ischaemic heart disease/angina was twofold higher in T2DM group compared with that in non-T2DM group. The relationship between T2DM and cardiovascular diseases reflects the impairments induced by T2DM on the cardiovascular system (Huang et al., 2017; Haas and McDonnell, 2018). The most prevalent concordant conditions among T2DM patients were hyperlipidemia, heart disease and atherosclerosis, with a greater prevalence in T2DM patients compared with that in non-T2DM individuals (Reunanen et al., 2000; Caughey et al., 2010; Huber et al., 2014; Chima et al., 2017). Another concordant comorbidity more likely to be recorded in T2DM patients was hyperuricemia/gout (OR 2.49, 95% CI, 2.45–2.53). As reported recently, there appears to be a three-way association between hyperuricemia, T2DM, and hypertension (Mortada, 2017). Moreover, hyperuricemia has emerged as an independent risk factor in T2DM development and hypertension through several postulated mechanisms (Kuwabara et al., 2017). The most prevalent discordant comorbidity in T2DM patients was GORD and peptic ulcer (67.9%), which was more than twice as likely to appear in T2DM patients than in non-T2DM individuals (OR 2.22, 95% CI, 2.20–2.24). A recent meta-analysis suggested that patients with T2DM are at a greater risk of GORD (Sun et al., 2015). Different hypotheses may be considered to justify this association, such as the higher prevalence of obesity and autonomic neuropathy among patients with T2DM (Frøkjær et al., 2007; De Vries et al., 2008). T2DM duration could also influence GORD and peptic ulcer symptoms, and could justify the higher prevalence of prescribed medication for this comorbidity in our cohort over 65 years of age (Kinekawa et al., 2008). Conversely, we must take into account that higher frequency of co-prescribing gastro-protective agents could be due to concomitant treatment with antiplatelet agents or anticoagulant medications rather than to the presence of gastroenteric diseases, as reported in studies carried out in Ireland and Australia using the RxRiskV Index to evaluate comorbidities in older T2DM patients (Caughey et al., 2010; O’Shea et al., 2013). During the study period, T2DM group showed a higher prevalence of comorbidities (concordant and discordant) with the exception for osteoporosis and corticosteroid-responsive disease. These results support the hypothesis of an Australian study by Caughey et al. (2010), who argued that these findings may infer an inadequacy in prescribing anti-osteoporosis medication level to older people with T2DM. A lower prevalence of corticosteroid-responsive disease was recorded in T2DM group. This finding could indicate restricted use of these drugs in T2DM due the predictable adverse effects of glucocorticoid therapy on blood glucose levels (Wallace and Metzger, 2018; Ceccarelli et al., 2019).

An increase in complexity in terms of comorbidity leads to increased costs. We showed that the average total annual cost due to concordant and discordant comorbidities was ∼70% higher in patients with T2DM than in people not suffering from T2DM. The worldwide economic impact of T2DM is well known (Giorda et al., 2011) because treatment of the complications associated with this disease is responsible for most of the management cost. Nevertheless, we highlighted that incremental cost for each comorbidity in addition to T2DM was related to concordant and discordant comorbidities. Therefore, one should not neglect discordant comorbidities in assessment of the cost associated with T2DM in future economic evaluations.

The comparison of cost among different countries is complex due to differences between healthcare systems. However, in agreement with several studies (Simpson et al., 2003; Bruno et al., 2012; Pagano et al., 2016), the greatest difference in cost of care between T2DM and non-T2DM groups was due to hospitalization. The influence of comorbidities on inpatient-care cost tended to be greater among patients with T2DM. This finding may also be because the duration of inpatient stay may increase with an increasing number of complications in T2DM patients (Jacobs et al., 1991). A higher percentage of patients who spent >3 days as hospital inpatients in the T2DM group than in the non-T2DM group was noted (12.3 vs. 8.0, respectively).

We also evaluated T2DM patients by categorizing them according to the cost distribution. We identified patients who were HC (i.e., above the 80th and above the 90th percentile). These patients were responsible for ∼80% of the use of healthcare services (primary care visits and/or hospital admissions and drug costs). These results are consistent with estimates reported by Zhang et al. (2017), who also suggested a significant skew in costs for T2DM patients. The high comorbidity-cost concentration indicated that it might be worthwhile to analyze patients requiring more expensive care by identifying, as Meyers et al., two subgroups of diabetic patients: those accruing for top 10th and those accruing for top 20th percentile of cost distribution (Meyers et al., 2014). These groups include all patients with significant economic and clinical burdens. Consistent with our analyses, other scholars have found that hospitalization accounted for almost all total spending among HC patients (Meyers et al., 2014; Rice et al., 2017; Nelson et al., 2019). Almost all of our HC patients were hospitalized at least once during 12-month follow-up, whereas Meyers and colleagues showed a lower (but explainable) figure because our study population was aged >65 years.

Our findings highlight the comorbidity score to be the strongest predictor of becoming an HC healthcare user: the higher is the number of comorbid conditions, the more costly and resource-consuming are patients. T2DM patients were significantly more likely to be in the top-10 percentile or the top-20 percentile of the total cost distribution if they had ≥5 comorbidities. This finding has enormous relevance considering that 50% of our T2DM patients had ≥5 comorbidities. A systematic review of the literature revealed that clinicians face a diverse range of challenges when dealing with multimorbid patients such as T2DM patients: fragmented healthcare services/systems; following multiple guidelines focusing on the management of a single condition; delivering patient-centered care; barriers to shared decision-making (Sinnott et al., 2013). Together with the comorbidity score, age, male sex and insulin treatment were the other markers of the cost of HC patients.

Our results emphasize the need for primary prevention through healthcare promotion and education. Moreover, the healthcare system should take into consideration the special needs of T2DM patients with comorbidities, and implement a multidisciplinary organization of care that can develop appropriate diagnostic and therapeutic strategies “tailored” to the specific needs of this group of patients. The heterogeneity of multimorbidity often necessitates a holistic and integrated approach to ensure that optimal care is provided for all co-existing conditions (The Academy of Medical Sciences, 2018). In fact, managing individual conditions separately may be ineffective and inefficient. Conversely, coordinated services can contribute to maximize healthcare efficiency and focus on the specific healthcare needs of each patient. When we analyzed the importance of each comorbidity in relation to a high cost, almost all of them contributed to the top decile of cost. Specifically, GORD and peptic ulcer, hyperlipidemia, cerebrovascular disease, and renal impairment were the more important conditions associated with a high cost; among them, the one with the greatest economic impact was renal impairment.

In agreement with the work of other scholars (An and Le, 2016; Lin et al., 2018), concordant and discordant conditions were more prevalent in T2DM patients and were associated with a higher cost of care. It is well known that disease-specific comorbidities represent an important economic burden in patients with T2DM. However, less attention has been paid to other types of comorbidities (Boyd et al., 2005). This factor must be considered particularly if T2DM patients are hospitalized for any of these conditions because the hospitalization cost represents the major determinant of the high cost, and is proportional to the duration of hospital stay. This, in turn, may be reduced by optimizing initiation of blood-glucose control, if possible, before hospital admission.

Our study had two main strengths. First, it had a population-based design within the Italian public-health system among T2DM patients over 65 years of age. Consequently, our data reflect a picture of comorbidities in T2DM and non-T2DM patients by identifying specific predictors of use of healthcare resources. Second, our study contributes to understanding of the determinants of an imbalanced distribution of comorbidity costs among T2DM patients. It also underlies the need to consider holistic medical care to better manage complex disease which determines high healthcare costs. In order to prevent negative consequences of T2DM in older patients, it would be necessary to provide some recommendations on lifestyle for patients who are below 65 years of age or who are suspected to become diabetics due to family history. The early identification of the suspected comorbidities can provide a framework for modifying the lifestyle in order to reduce the ultimately cost of therapy as well as to improve the quality of life despite of T2DM disease.

Our study had three main limitations. The first was the nature of the Italian administrative database used to obtain data. Although powerful tools, pharmaceutical records do not provide information about private-practice prescriptions and out-of-pocket expenditure. Thus, the prevalence of some diseases reported in our analysis may have been underestimated. However, patients who take drugs long-term are unlikely to buy them over-the-counter. The second limitation was the lack of information relating to the causal relationship between patient characteristics and healthcare costs among T2DM patients. Third, we limited our cohorts to patients over 65 years of age, so the results apply strictly to this age group.

## Conclusion

We demonstrated a greater prevalence of most concordant and discordant T2DM-related comorbidities and the associated cost in older patients compared with those not suffering from T2DM. HC T2DM patients accrued >80% of the total cost for comorbidities, and this cost increased in parallel with an increasing number of comorbidities. Our study strengthens the importance of implementing integrated care models, which include a holistic and patient-tailored focus, to achieve more efficacious T2DM care in the context of the growing proportion of multimorbidity in the older population. Moreover, it underlines the need to reduce the number of hospitalizations and duration of hospital stay due to T2DM. Among other factors, this can be achieved by intensifying control of blood glucose before hospital admission for elective procedures. Finally, this study can be a useful tool for healthcare stakeholders when planning future interventions to track and reduce the cost of T2DM-related disease.

### Contribution to the Field

T2DM prevalence has increased dramatically worldwide to become an important healthcare concern, occurring particularly in those aged ≥65 years. Furthermore, the resulting health and economic T2DM impacts go beyond the condition itself because diabetic patients are subject to disabling complications and incur very high costs to the Italian National Health Service. To date, little is known about the specific comorbidities contributing to higher costs in patients with T2DM, particularly in older patients. The retrospective cohort study proposed here describes the prevalence, type, and cost of comorbidities occurring in older patients with T2DM and correlation with high cost patients. It therefore seems that there is a greater prevalence of most T2DM-related comorbidities and the associated cost in older patients compared with those not suffering from T2DM. Hence, it appears that HC T2DM patients accrued >80% of the total cost for comorbidities, and this cost increased in parallel with an increasing number of comorbidities. These findings reflect the importance of implementing integrated care models, which include a holistic and patient-tailored focus, to achieve more efficacious T2DM care in the context of the growing proportion of multimorbidity in the older population.

## Data Availability Statement

The raw data supporting the conclusions of this article will be made available by the authors, without undue reservation.

## Ethics Statement

Ethical review and approval was not required for the study on human participants in accordance with the local legislation and institutional requirements. Written informed consent for participation was not required for this study in accordance with the national legislation and the institutional requirements.

## Author Contributions

IAG, EM, VO, and AG-M conceived the study. IG conducted the study. IG, VM, VO, and EM analyzed the results and wrote the original draft. BP, SM, OV, MF, AP-T, GR, and EM reviewed the manuscript. All authors agreed with the final version of the manuscript.

## Conflict of Interest

The authors declare that the research was conducted in the absence of any commercial or financial relationships that could be construed as a potential conflict of interest.
