# Supplementary material for: Comorbidity in an Older Population with Type-2 Diabetes Mellitus: Identification of the Characteristics and Healthcare Utilization of High-Cost Patients
Source: Front Pharmacol. 2020 Nov 30;11:586187. doi: 10.3389/fphar.2020.586187 (PMC7970761; doi:10.3389/fphar.2020.586187)
Supplement: Supplementary file 1 [file datasheet1.docx]

Supplementary Material

# Supplementary Tables

**Supplementary Table 1.** CaReDB characteristics.

| Characteristic |  |
| --- | --- |
| Geographic area | Campania, Italy |
| Population covered | ~5.8 million inhabitants |
| Age-span covered | Whole population |
| Time-span covered ^a^ | 2010–2019 |
| Scope | Drug utilisation and outcome research; real-world evidence for public health; pharmacoepidemiologic and pharmacoeconomic analyses |
| Data sources | Specific fields in the data sources contributing to a database |
| Demographic information | Patient ID; sex; date of birth; municipality of residence; district; local health unit |
| Outpatient pharmacy records | Patient ID; drug code; prescription date; delivery date; quantity; ATC code; price; defined daily dose; drug distribution channel (file F, file D) |
| Hospital-discharge records | Patient ID; type of admission; date of admission; reasons for discharge; diagnoses (ICD-9 code); procedures (ICD-9 code); date of discharge; disease-related group |
| Type of codes for diagnoses | Hospital: ICD-9-CM |
| Type of codes for medications | ATC classification |

ATC=Anatomical Therapeutic Chemical; ICD-9-CM=International Classification of Diseases, 9th Revision, Clinical Modification.

^a^Time-span covered was 2009–2018 for hospital-discharge records, and 2014–2019 for outpatient pharmacy records.

**Supplementary Table 2.** List of ATC codes used in the RxRiskV (mod).

| **Disease** | **ATC code** |
| --- | --- |
| Alcohol dependency | N07BB03, N07BB04, N07BB01 |
| Allergies | R01AC, R01AD, R06AD02, R06AD03, R06AD04, R06AD05, R06AD06, R06AD07, R06AD08, R06AD09, R06AD52, R06AD55, R06AE, R06AK, R06AX, excluding R06AX27 R06AX28 R06AX53 R06AX58 |
| Anti-coagulation therapy | B01AA03 B01AA04, B01AA07 – B01AA11, B01AB01, B01AB02, B01AB04–B01AB06, B01AB10, B01AE07, B01AF01, B01AF02 |
| Anti-platelet therapy | B01AC04 – B01AC19, B01AC30, B01AC22, B01AC23, B01AC24, B01AC27 |
| Anxiety | N05BA01 – N05BA12, N05BB01 |
| Arrhythmia | C01AA05, C01BA01–C01BD01, C07AA07 |
| Benign prostatic hypertrophy | G04CA01–G04CA04, G04CB01 |
| Bipolar disorder | N05AN01 |
| Chronic airways disease | R03AC, R03AK, R03AL01– R03AL09, R03BA, R03AB, R03BC01, R03BC03, R03BX01, R03CA02, R03CB, R03CC, R03CC53, R03DA, R03DB, R03DC, R03DX05, R03BB |
| Dementia | N06DA02, N06DA03, N06DA04, N06DX |
| Depression | N06A |
| Epilepsy | N03AA01–N03AA04, N03AA30, N03AB01–N03AB05 N03AB52, N03AB54, N03AC01, N03AC02, N03AC03, N03AD01, N03AD02, N03AD03, N03AD51, N03AE01, N03AF01, N03AF02, N03AG01, N03AG02, N03AG03, N03AG04, N03AG05, N03AG06, N03AF03, N03AF04, N03AX |
| Glaucoma | S01EA01, S01EA02, S01EA03, S01EA04, S01EA05, S01EA51, S01EB01, S01EB02, S01EB03, S01EC03, S01EC04, S01EC54, S01ED01, S01ED02, S01ED03, S01ED04, S01ED05, S01ED06, S01ED51, S01ED52, S01ED54, S01EE01, S01EE02, S01EE03, S01EE04, S01EX01, S01EX02 |
| GORD and peptic ulcer | A02B |
| Heart disease | C07AA01, C07AA02, C07AA03, C07AA05, C07AA06, C07AA07, C07AA12, C07AA14, C07AA15, C07AA16, C07AA17, C07AA19, C07AA23, C07AA27, C07AA57, C07AB, C07AG01, C07AG02, C07BA02, C07BA05, C07BA06, C07BA07, C07BA12, C07BA68, C07BB02, C07BB03, C07BB04, C07BB06, C07BB07, C07BB52, C07BG01, C07CA02, C07CA03, C07CA17, C07CA23, C07CB02, C07CB03, C07CB53, C07CG01, C07DA06, C07DB01, C07FA05, C07FB02, C07FB03, C07FB07, C08CA01, C08CA02, C08CA03, C08CA04, C08CA05, C08CA06, C08CA07, C08CA08, C08CA09, C08CA10, C08CA11, C08CA12, C08CA13, C08CA14, C08CA15, C08CA55, C08CX01, C08DA01, C08DA02, C08DA51, C08DB01 |
| Hepatitis-C virus | J05AB54 |
| Human immunodeficiency virus | J05AE01–J05AE08, J05AF01–J05AF11, J05AG01, J05AG02, J05AG03 J05AR01–J05AR06, J05AX07 |
| Hyperkaliemia | V03AE01 |
| Hyperlipidemia | C10AA, C10AB, C10AC, C10AD, C10AX, C10BA, C10BX |
| Hypertension | C03AA, C03AB, C03AH, C03AX01, C02CA04, C03BA02, C03BA03, C03BA04, C03BA05, C03BA07, C03BA08, C03BA09, C03BA10, C03BA11, C03DB01, C03DB02, C03EA, C09BA02, C09BA03, C09BA04, C09BA05, C09BA06, C09BA07, C09BA08, C09BA09, C09BB, C09DB, C09DA01, C09DA02, C09DA03, C09DA04, C09DA06, C09DA07, C09DA08, C02AB01, C02AB02, C02AC01, C02AC02, C02AC04, C02AC05, C02DB02, C02DB03, C02DB04, C02DC01, C02DD01, C02DG01, C02KA01, C02KB01, C02KC01, C02KD01, C02KX01, C09XA |
| Hyperuricemia/gout | M04AA01, M04AA02, M04AA03, M04AA51, M04AB01, M04AB02, M04AB03, M04AB04, M04AB05, M04AC01 |
| Hypothyroidism | H03AA01, H03AA02 |
| Inflammatory arthritis | M04AA01, M04AA02, M04AA03, M04AA51, M04AB01, M04AB02, M04AB03, M04AB04, M04AB05, M04AC01 |
| Inflammatory bowel disease | A07EC01, A07EC02, A07EC03, A07EC04 |
| Inflammatory/pain | M01AB, M01AC01, M01AC02, M01AC04, M01AC05, M01AC06, M01AE, M01AG, M01AH, N02BE51, NO2BA01, N02BG06 |
| Ischaemic heart disease/angina | C01DA02, C01DA04, C01DA05, C01DA07, C01DA08, C01DA09, C01DA13, C01DA14, C01DX14, C01DX16, C01EB15, C01EB17, C01EB18 |
| Malignancies | L01AA01, L01AA02, L01AA03, L01AA05, L01AA06, L01AA07, L01AA08, L01AB, L01AC, L01AD, L01AG01, L01AX L01BA01, L01BA03, L01BA04, L01BB02, L01BB03, L01BB04, L01BB05, L01BB06, L01BB07, L01BC, L01CA, L01CB, L01CC01, L01CD L01CX01 L01DA01 L01DB, L01DC, L01XA, L01XB01, L01XC, L01XD01, L01XD03, L01XD04 L01XD05 L01XD06 L01XE, L01XX, L02BA01, L02BA02, L02BG02, L02BG03, L02BG04, L02BG06, L02BB01, L02BB03, L02AE02, L02AE04, L02AB01 |
| Migraine | N02CA01, N02CA02, N02CA04, N02CA07, N02CA51 N02CA52, N02CA72 N02CB01, N02CC01, N02CC02, N02CC03, N02CC04, N02CC05, N02CC06, N02CC07, N02CX01 |
| Osteoporosis | M05BA, M05BB, M05BX03, G03XC01, A12AX92, H05AA02 |
| Pain (opiates) | N02AA, N02AB, N02AC01, N02AC03, N02AC04, N02AC05, N02AC52, N02AC54, N02AC74, N02AD01, N02AD02, N02AE01, N02AF01, N02AF02, N02AG, N02AJ06, N02AJ08, N02AJ17, N02AX01, N02AX02, N02AX52, NO2AX05 |
| Pancreatic insufficiency | A09AA02 |
| Parkinson’s disease | N04AA01, N04AA02, N04AA03, N04AA04, N04AA05, N04AA08, N04AA09, N04AA10, N04AA11, N04AA12, N04AB01, N04AB02, N04AC01, N04AC30, N04BA01, N04BA02, N04BA03, N04BA04, N04BA05, N04BA06, N04BB01, N04BC01, N04BC02, N04BC03, N04BC04, N04BC05, N04BC06, N04BC07, N04BD01, N04BX01, N04BX02, N0BC09, N04BD02 |
| Psoriasis | D05BB01, D05BB02, D05AX |
| Psychotic illness | N05AA, N05AB, N05AC, N05AD, N05AE, N05AF, N05AG N05AH, N05AL, N05AN01, N05AX |
| Smoking cessation | N06AX12, N07BA01, N07BA03 |
| Corticosteroid-responsive disease – systemic corticosteroid use | H02AB, H02AA |
| Transplant | L04AA01, L04AA02, L04AA03, L04AA04, L04AA05, L04AA06, L04AA08, L04AA09, L04AA10, L04AA11, L04AA12, L04AA14, L04AA15, L04AA16, L04AA17, L04AA18, L04AA19, L04AA21, L04AD01, L04AD02, L04AX01 |
| Tuberculosis | J04AB04, J04AB05, J04AB30, J04AC01, J04AC51, J04AD01, J04AD02, J04AD03, J04AK01, J04AK02, J04AM02, J04AM05 |

**Supplementary Table 3.** ICD–9 codes for micro/macrovascular complications.

| **Complication** | **Clinical condition** | | **ICD-9-CM diagnosis** | **Procedure code** |
| --- | --- | --- | --- | --- |
| **Macrovascular** | Cardiovascular disease | - Ischemic heart disease - Acute myocardial infarction - Arrhythmia - Congestive heart failure - Atherosclerosis | 410.xx (excluding 410.x1)  411.xx, 413.xx, 414.xx | 36.0x, 36.1x,36.2, 36.3,  36.9, 88.5x,37.8x |
|  |  |  | 410.x1, 427.5  412* |  |
|  |  |  | 426.xx, 427.0, 427.1,  427.2, 427.3, 427.4,  427.6x, 427.8x, 427.9 |  |
|  |  |  | 428.x, 785.5x, 402.01,  402.11, 402.91, 404.01,  404.03, 404.11, 404.13,  404.91, 404.93 |  |
|  |  |  | 429.2, 440.xx | 38.12 |
|  | Peripheral vascular  disease | - Peripheral vascular disease - Lower-limb complications | 250.7x, 441.xx, 442.xx,  443.xx, 444.xx, 447.1 | 38.13, 38.14, 38.16,  38.18, 39.25, 39.29,  39.50, 39.90, 99.10 |
|  |  |  | 040.0, 681.1x, 682.6,  682.7, 707.1x, 711.9,  713.5, 730.0, 730.0x  730.1x 730.2x 730.3x  785.4 | 84.10-84.19, 86.22 |
|  | Cerebrovascular disease | | 430.xx-438.xx | 38.11, 38.12 |
| **Microvascular** | Nephropathy | | 250.4x, 403.xx, 404.xx,  581.81, 584.x, 585, 586,  593.9 | 38.95, 39.27, 39.42,  39.95, 54.93, 54.98 |
|  | Neuropathy | | 250.6x, 337.1, 354, 355,  357.2, 377.xx, 536.3,  596.54, 607.84, 713.5 |  |
|  | Retinopathy | | 250.5x, 361.0x, 361.9,  362.0x, 362.1x, 362.83,  364.42, 365.44, 365.6x,  366.1x, 366.41, 369.xx,  377.xx, 379.2x, 369.0x-  369.9x, V431 | 13.xx, 14.33,14.34,  14.35,  14.53, 14.54, 14.55,  14.73, 14.74 |

ICD-9-CM=International Classification of Disease, 9^th^ edition, clinical modification.
